# Prospective population pharmacokinetic study of tacrolimus in adult recipients early after liver transplantation: A comparison of Michaelis-Menten and theory-based pharmacokinetic models

**DOI:** 10.3389/fphar.2022.1031969

**Published:** 2022-11-09

**Authors:** Xiao-Jun Cai, Rui-Dong Li, Jian-Hua Li, Yi-Feng Tao, Quan-Bao Zhang, Cong-Huan Shen, Xiao-Fei Zhang, Zheng-Xin Wang, Zheng Jiao

**Affiliations:** ^1^ Department of Pharmacy, The Affiliated Wuxi People’s Hospital of Nanjing Medical University, Wuxi, China; ^2^ Department of Pharmacy, Huashan Hospital, Fudan University, Shanghai, China; ^3^ Department of General Surgery, Huashan Hospital, Fudan University, Shanghai, China

**Keywords:** tacrolimus, liver transplantation, population pharmacokinetics, nonlinear pharmacokinetics, Monte Carlo simulation

## Abstract

**Background and Objective:** Tacrolimus, a calcineurin inhibitor widely used as a potent immunosuppressant to prevent graft rejection, exhibits nonlinear kinetics in patients with kidney transplantation and nephrotic syndrome. However, whether nonlinear drug metabolism occurs in adult patients undergoing liver transplantation remains unclear, as do the main underlying mechanisms. Therefore, here we aimed to further confirm the characteristics of nonlinearity through a large sample size, and determine the potential influence of nonlinearity and its possible mechanisms.

**Methods:** In total, 906 trough concentrations from 176 adult patients (150 men/26 women; average age: 50.68 ± 9.71 years, average weight: 64.54 ± 11.85 kg after first liver transplantation) were included in this study. Population pharmacokinetic analysis was performed using NONMEM^®^. Two modeling strategies, theory-based linear compartmental and nonlinear Michaelis–Menten (MM) models, were evaluated and compared. Potential covariates were screened using a stepwise approach. Bootstrap, prediction-, and simulation-based diagnostics (prediction-corrected visual predictive checks) were performed to determine model stability and predictive performance. Finally, Monte Carlo simulations based on the superior model were conducted to design dosing regimens.

**Results:** Postoperative days (POD), Aspartate aminotransferase (AST), daily tacrolimus dose, triazole antifungal agent (TAF) co-therapy, and recipient CYP3A5*3 genotype constituted the main factors in the theory-based compartmental final model, whereas POD, Total serum bilirubin (TBIL), Haematocrit (HCT), TAF co-therapy, and recipient CYP3A5*3 genotype were important in the nonlinear MM model. The theory-based final model exhibited 234 L h^−1^ apparent plasma clearance and 11,000 L plasma distribution volume. The maximum dose rate (*V*_*max*_) of the nonlinear MM model was 6.62 mg day^−1^; the average concentration at steady state at half-*V*_*max*_ (*K*_*m*_) was 6.46 ng ml^−1^. The nonlinear MM final model was superior to the theory-based final model and used to propose dosing regimens based on simulations.

**Conclusion:** Our findings demonstrate that saturated tacrolimus concentration-dependent binding to erythrocytes and the influence of daily tacrolimus dose on metabolism may partly contribute to nonlinearity. Further investigation is needed is need to explore the causes of nonlinear pharmacokinetic of tacrolimus. The nonlinear MM model can provide reliable support for tacrolimus dosing optimization and adjustment in adult patients undergoing liver transplantation.

## 1 Introduction

Tacrolimus, a potent calcineurin inhibitor, constitutes the cornerstone of most immunosuppressive regimens for solid organ transplantation (Bentata, 2020). It is highly lipophilic and poorly soluble, with a mean bioavailability of 25% (4–89%) (Jusko et al., 1995). Orally administered tacrolimus is rapidly absorbed, reaching peak concentration after 0.5–1 h, and extensively binding to erythrocytes and albumin (approximately 99%) (Yu et al., 2018). Subsequently, it undergoes extensive first-pass metabolism, primarily *via* the cytochrome P450 isoenzymes CYP3A4 and CYP3A5 and the efflux pump P-glycoprotein (P-gp) (Vanhove et al., 2016). Finally, tacrolimus is fully converted into metabolites that are mainly excreted through bile (>95%) into the feces, with <1% of the parent drug remaining unchanged in the urine or feces (Moller et al., 1999; Staatz and Tett, 2004).

Tacrolimus exhibits a narrow therapeutic index and large intra- and inter-individual pharmacokinetic (PK) variability in liver transplant recipients owing to multiple factors (Coste and Lemaitre, 2022), including postoperative time (POD), patient demographics, analytical assay type, daily tacrolimus dose (DD), graft type (whole or split-liver), hepatic function, concomitant food intake, gastrointestinal disorders, drug–drug interactions, and genetic factors (Christians et al., 2002; Van Boekel et al., 2012; Campagne et al., 2019), especially single-nucleotide polymorphisms in *CYP3A* genes (i.e., CYP3A5*3 (rs776746) and CYP3A4*1G (rs2242480) alleles) (Andreu et al., 2017; Dong et al., 2022). Small ubiquitin-like modifier 4 (SUMO4) directly or indirectly regulates the CYP3A5 enzyme through the NF-κB signaling pathway, and a *SUMO4* (rs237025) genetic variant is associated with a higher tacrolimus dose-corrected concentration (*C*_*0*_ *D* ^−1^) early after liver transplantation (Zhang et al., 2018). Such increased tacrolimus exposure is primarily regulated by *NR112* (rs2276707), which encodes a nuclear receptor that regulates CYP3A and drug transporter expression (Kliewer et al., 2002; Barraclough et al., 2012).

This variability affords higher risk for poor long-term outcomes, such as late allograft rejection, graft loss, adverse effects (e.g., nephrotoxicity and hypertension), and death despite transplant function (Lieber and Volk, 2013; Alissa et al., 2022). To optimize therapeutic efficacy and minimize tacrolimus-induced toxicity, therapeutic drug monitoring (TDM) and population pharmacokinetic (popPK) models are widely used to guide personalized tacrolimus dosing and ensure target whole-blood trough concentrations (*C*_*0*_), especially early post-transplantation (De Jonge et al., 2009; Campagne et al., 2019).

We previously reported nonlinear tacrolimus kinetics in adult patient populations receiving liver and kidney transplantations (Zhao et al., 2016; Cai et al., 2020), similar to results in pediatric patients with primary nephrotic syndrome (PNS) (Huang et al., 2020). Moreover, tacrolimus PK nonlinearity differed among solid organ transplantations (Cai et al., 2020). Such nonlinearity may partly derive from its poor aqueous solubility (1–2 μg ml^−1^) (Lee et al., 2016) and low intestinal membrane permeability (Tamura et al., 2002), leading to dissolution rate-limited absorption in the gut and variable and low oral bioavailability. Additionally, saturated tacrolimus concentration-dependent binding to erythrocytes and albumin might facilitate nonlinear PK behavior, especially regarding drug distribution (Chow et al., 1997). The TDM effect may also engender nonlinearity, as higher drug clearance is usually associated with lower drug concentrations, leading to higher prescribed doses. Consequently, TDM induces a correlation between total DD and clearance. This may be classified as nonlinear with sampling below three dose levels (Ahn et al., 2005).

Therefore, the present study aimed to 1) further confirm tacrolimus nonlinearity in liver transplant recipients and investigate possible nonlinear mechanisms by expanding the sample size and applying two modeling strategies, theory-based linear compartmental and nonlinear Michaelis–Menten (MM) models, and comparing their differences; 2) further identify any variability, including genetic information of tacrolimus PK, based on routine TDM data prospectively collected at Huashan Hospital to facilitate dose individualization; and 3) propose initial dosing regimens early after adult liver transplantation *via* Monte Carlo simulations based on the superior model.

## 2 Materials and methods

### 2.1 Subjects and clinical data collection

A total of 176 adult recipients (150 men/26 women) who underwent their first liver transplantation using organs donated after cardiac death and received immediate-release oral tacrolimus capsules (Prograf, Astellas, Dublin, Ireland) at Huashan Hospital, Fudan University, from June 2018 to October 2019, were included. Patient follow-up was conducted until the day of post-surgery discharge. To ensure tacrolimus concentrations at or near the steady state, records were retrieved only after ≥3 repeated oral doses administered at consistent dose rates. Exclusion criteria included severe gastrointestinal disorders, acute rejection, or secondary liver transplantation.

Tacrolimus *C*_*0*_, laboratory test results, and concomitant medications were prospectively acquired. Patient demographic characteristics, such as body weight, height, age, sex, grafted hepatic weight, dosing regimen, and sampling time, were collected. The study protocol was approved by the Ethics Committee of Huashan Hospital, Fudan University, and registered at the Center for Clinical Research and Biostatistics (www2.ccrb.edu.hk, No: CUHK_TMP00250). Written informed consent was obtained from all subjects. This study was conducted in accordance with the Declaration of Helsinki (2013).

### 2.2 Immunosuppressive therapy

All patients received post-transplantation immunosuppressive therapy with tacrolimus and steroids. The tacrolimus dosage was initially 0.5–1 mg every 12 h (q12 h) and then empirically adjusted to achieve steady-state C_0_ within 8–12, 8–10, and 6–8 ng ml^−1^ in the first 3, between 3 and 6, and subsequent postoperative months, respectively.

Intravenous methylprednisolone (500 mg) was administered on the operative day, followed by 80 mg q12 h on postoperative days 1–3, tapering to 80, 40, and 20 mg day^−1^ on postoperative days 4–5, 6–7, and 8–10, respectively. Oral prednisolone (12 mg day^−1^) was started on postoperative day 11 and tapered to 4 mg day^−1^ at a rate of 4 mg day^−1^, except for patients with hepatocellular carcinoma who underwent liver transplantation. During the second postoperative month, corticosteroid-free treatment was administered, except for patients with autoimmune hepatitis. Mycophenolate mofetil (CellCept, Roche Pharma Ltd., Shanghai, China) was administered orally q12 h at 0.5 g day^−1^ to recipients exhibiting a glomerular filtration rate below 60 ml min^−1^ per 1.73 m^2^.

### 2.3 Blood sample collection and bioassay

Whole blood samples were drawn before the morning dose to measure C_0_ using an enzyme-multiplied immunoassay technique (SYVA Viva-Emit 2000 kit, Siemens Healthcare Diagnostics Inc., Germany). The coefficient of variation of intra- and inter-day precision was within 10% (calibration range 2.0–30 ng ml^−1^).

### 2.4 Genotyping

Ethylenediaminetetra acetic acid-anticoagulated whole blood obtained from liver transplant recipients and their corresponding donors was stored at −20°C. Genotyping of four single-nucleotide polymorphisms: CYP3A5*3 (rs776746), CYP3A4*1G (rs2242480), *SUMO4* (rs237025), and *NR112* (rs2276707) was performed by an independent external contractor (Sangon Biotechnology Co., Ltd., Shanghai, China) using a DNA direct sequencing analyzer (Applied Biosystems 3730XL, Foster City, CA, United States). Allele and genotype frequencies were analyzed using the online software SHEsis (http://analysis.bio-x.cn/myAnalysis.php). Hardy–Weinberg equilibrium was assessed using Pearson’s chi-squared test. Appendix S1 includes gene amplification and sequencing details.

### 2.5 Population pharmacokinetic analysis

PopPK analysis was performed using nonlinear mixed-effects modeling software (NONMEM^®^, version 7.4; ICON Development Solutions, Ellicott City, MD, United States) compiled with gfortran 4.6.0 and interfaced with Perl-speaks-NONMEM (version 4.7.0; uupharmacometrics.github.io/PsN). The NONMEM output was analyzed using R software (version 3.5.1; www.r-project.org). First-order conditional estimation methodology with interaction between interpatient and residual variability was employed for model development.

#### 2.5.1 Base model

Two modeling strategies, theory-based linear compartmental and nonlinear MM empirical modeling, were employed in model development. In theory-based modeling strategies, the whole blood concentration (*C*_*wb*_) of tacrolimus is converted into plasma concentration (*C*_*p*_) because of saturated binding to red blood cells. The theory-based linear PK model was a one-compartment model with first-order absorption and elimination and was parameterized in terms of apparent total plasma clearance (*CL*_*pl*_/*F*), apparent plasma distribution volume (*V*_*pl*_ */F*), and absorption rate constant (*K*_*a*_). As no sampling was performed during the absorption phase, *Ka* was fixed at 4.48 h^−1^ based on published data (Zhu et al., 2015).

The nonlinear MM empirical formula is shown as follows:

$$MM\;model:Dose=\frac{Vm\times C_{0}}{Km+C_{0}}$$ (1)

where *Dose* is the daily oral tacrolimus dose, *V*_*m*_ is the maximum steady-state dosing rate (DD) (mg day^−1^), and *K*_*m*_ is an MM constant equal to the steady-state concentration at the half-maximum dose rate (ng ml^−1^). C_0_ represents steady-state values for at least three oral doses.

Inter-subject variability (ISV) in PK parameters, except *Ka*, is described by the exponential model below:

$$P_{i}=TV\left(P\right)\times \exp\left(\eta i\right)$$ (2)

where *P*_*i*_ is the PK parameter estimation of the *i* ^*th*^ subject and *TV*(*P*) is the typical value of the population parameter. *η*_*i*_ is defined as the symmetrically distributed ISV (mean = 0; variance = *ω*_*i*_^*2*^).

Residual unexplained variability (RUV) was tested using additive (Eq. 3), proportional (Eq. 4), and combination error (Eq. 5) models.

$$Y=F+\epsilon_{1}$$ (3)

$$Y=F+F\times \epsilon_{1}$$ (4)

$$Y=F+F\times \epsilon_{1}+\epsilon_{2}$$ (5)

where *Y* is the observed concentration, *F* is the individual predicted concentration, and *ε*_*n*_ represents the symmetrically distributed random variability (mean = 0; variance = *σ*_*n*_^*2*^).

#### 2.5.2 Covariate model

The influence of potential covariates on tacrolimus PK variability, including age, sex, height, body weight (WT), fat-free mass, grafted hepatic weight, hematocrit (HCT), albumin (ALB), aspartate aminotransferase (AST), total serum bilirubin (TBIL), creatinine clearance, POD, concomitant medications, and CYP3A5*3, CYPP3A4*1G, SUMO4, and *NR112* genetic polymorphisms in both donors and recipients, was investigated. The daily tacrolimus dose was screened only for the theory-based linear compartmental model. Only co-medications with a proportion >10% in all patients were tested. These covariates were selected as clinically plausible.

The potential influence of nonlinearity and the functional forms of covariates on model predictability were tested using two modeling strategies.

##### 2.5.2.1 Strategy I: Theory-based linear compartmental model

A theory-based linear compartmental model was developed based on well-accepted theoretical relationships. Tacrolimus extensively binds to erythrocytes, albumin, and a1-acid glycoprotein (>99%; <1% remaining unbound) (Yu et al., 2018). Free tacrolimus concentration depends on its affinity to plasma proteins and erythrocytes. HCT-standardized concentrations maintain a stable ratio with therapeutically active unbound concentrations (assumed to be proportional to *C*_*p*_). To eliminate the influence of confounding factors of HCT changes on predicting tacrolimus concentration, the *C*_*wb*_ was converted into *C*_*p*_. PK disposition parameters were estimated from model-predicted *C*_*p*_ rather than measured *C*_*wb*_ under the assumption that tacrolimus binds linearly to plasma components but nonlinearly (strongly) to erythrocytes (Storset et al., 2014a).

Similar to published descriptions (Storset et al., 2014a; Van Erp et al., 2016), tacrolimus *C*_*p*_ was estimated as follows:

$$C_{\text{wb}}=C_{p}+\frac{C_{p}\times \text{HCT}\times B_{\max}}{C_{p}+K_{D}}$$ (6)

where *C*_*wb*_ and *C*_*p*_ are given in ng mL^−1^, and HCT as %. *B*_*max*_ is the maximum drug concentration that can be bound per unit volume of red blood cells equal to 418 μg L^−1^, *K*_*D*_ is a dissociation equilibrium constant equal to 3.8 μg L^−1^ (Jusko et al., 1995; Storset et al., 2014a).

Therefore, all parameter estimates are expressed as plasma PK parameters and the model can predict both *C*_*wb*_ and *C*_*p*_, provided that HCT is known.

##### 2.5.2.2 Strategy II: Nonlinear Michaelis–Menten empirical model

Covariate influence on the MM constant (*K*_*m*_) was empirically investigated. As the tacrolimus steady-state PK changed with time after transplantation and the cut-off point was approximately 10 days after surgery, time factors (10/*θ*, Eq. 7) were introduced to investigate whether the MM model with time-variant *K*_*m*_ was superior.

$$\text{DD}\left(mg\;{day}^{-1}\right)=\frac{V_{m}\times C_{0}}{\frac{10}{\theta }\times K_{m}+C_{0}}$$ (7)

where *θ* = POD if 0 < POD≤10, or = 10 if POD >10.

Continuous covariates were normalized to the population median values and modeled using linear (Eq. 8), exponential (Eq. 9), and power (Eq. 10) models.

$$P_{i}=TV\left(P\right)+\theta_{cov}\times \left(COV_{i}/COV_{median}\right)$$ (8)

$$P_{i}=TV\left(P\right)\times \exp\left(COV_{i}/COV_{median}\times \theta_{cov}\right)$$ (9)

$$P_{i}=TV\left(P\right)\times {\left(COV_{i}/COV_{median}\right)}^{\theta_{cov}}$$ (10)

where *COV*_*i*_ is the covariate value of the *i* ^*th*^ individual, *COV*_*median*_ is the population median value of the covariate, and *θ*_*cov*_ is the coefficient term of the covariate effect to be estimated.

The binary covariates, such as concomitant medications, were tested using a scale model (Eq. 11).

$$P_{i}=TV\left(P\right)\times \left(1+\theta_{cov}\times COV_{i}\right)$$ (11)

where *TV(P)* is the typical value for parameter *P* without co-therapy with azole antifungal agents (*COV*_*i*_ = 0) and *θ*_*cov*_ is the fractional change in parameter *P* with co-therapy (*COV*_*i*_ = 1). For the effect of genetic polymorphism, *TV(P)* is the typical value for parameter *P* for the wild-type genotype (*COV*_*i*_ = 0), and *θ*_*cov*_ is the fractional change in parameter *P* for the heterozygous (*COV*_*i*_ = 1) or mutant (*COV*_*i*_ = 2) genotypes. Mutant and heterozygous genotypes can also be grouped together (*COV*_*i*_ = 1) as a binary covariate for analysis.

Covariates were screened in a stepwise manner, with forward inclusion and backward elimination. Individual variable effect on the parameters was tested using the likelihood ratio. For forward inclusion, an objective function value (OFV) reduction of at least 3.84 for 1 degree of freedom (df) (χ^2^ test, *p* < 0.05, df = 1) was used as a criterion for covariate inclusion. For backward elimination, an OFV increase ≥6.63 (χ^2^ test, *p* < 0.01, df = 1) served as a criterion for covariate retention.

Covariates without pharmacological or biological plausibility or with <20% effect on a parameter were not retained. Improved parameter estimation precision and goodness-of-fit (GOF) plots, reduced ISV and RUV, and parameter estimate stability were also used to select covariates. Shrinkage extent was evaluated using the final model.

#### 2.5.3 Model evaluation

Prediction- and simulation-based diagnostics were conducted to compare the predictive performances of the two models. GOF plots, including observed concentrations (OBS) *versus* population prediction (PRED), individual prediction (IPRED), conditional weighted residuals (CWRES) *versus* PRED, and time-after-dose (TAD) were used to evaluate the fit of the final model to the data.

PREDs and IPREDs were estimated and compared to the corresponding OBS based on the relative prediction error (PE%, Eq. 12) and individual prediction error (IPE%, Eq. 13), respectively.

$$PE\left(\%\right)=\frac{PRED-OBS}{OBS}\times 100\%$$ (12)

$$IPE\%=\frac{IPRED-OBS}{OBS}\times 100\%$$ (13)

The median prediction error (MDPE%) and median absolute prediction error (MAPE%) were used to test predictive performance accuracy and precision, respectively. PE% within ±20% (*F*_*20*_) and 30% (*F*_*30*_), indices of both accuracy and precision, were also calculated. Models with lower MDPE and MAPE values and less PE% beyond ±20 and ±30% were considered superior. *IF*_*20*_ and *IF*_*30*_, indicating IPE% within ±20% and ±30%, respectively, were also used as a combination index of both accuracy and precision.

Additionally, nonparametric bootstrap, prediction- and variability-corrected visual predictive checks (pvcVPCs), and normalized prediction distribution errors (NPDEs) were employed to assess the candidate final model. For the nonparametric bootstrap procedure, 1,000 replicate bootstrap datasets were generated by random resampling from the raw database and fitted with the same model to obtain parameter estimates for each replicate. The medians and 2.5^th^–97.5^th^ percentiles of the parameters after bootstrap runs with successful convergence were compared with the final model parameter estimates.

For the pvcVPCs, 1,000 new datasets were simulated in NONMEM using the final model to simulate the expected concentrations. The concentration–time profiles were plotted for the 50th, 10th, and 90th percentiles of the simulated data and overlaid with the observed data.

NPDE with 2000 simulations was performed for each observation in the raw dataset using the final model. The NPDE results were summarized statistically and graphically using the NPDE add-on package in R (version 2.0; www.npde.biostat.fr). Their distributions were evaluated to test whether the final model fully described the observed data; plots of NPDEs *versus* observations and time were also investigated.

### 2.6 Model-informed individualized dosing

Given how crucial is the target tacrolimus C_0_ (8–12 ng ml ^−1^) in the first week post-transplantation, Monte Carlo simulations were performed to optimize the starting dosing regimen based on the established final popPK model and identified covariates to achieve the target tacrolimus *C*_*0*_ after 7 days multiple oral doses. From 200 simulations performed using the initial dataset, the steady-state *C*_*0*_ of each simulated subject was calculated.

## 3 Results

### 3.1 Study population

A total of 906 tacrolimus *C*_*0*_ values obtained from 176 liver transplant patients were prospectively collected during hospitalization of eligible patients. Table 1 shows the main demographic characteristics of the enrolled patients. Each patient had a median of five tacrolimus observations (mean tacrolimus dose: 2.73 ± 1.56 mg day^−1^; corresponding median *C*_*0*_: 5.96 (0.30–23.11) ng ml^−1^. Concentrations below lower quantification limits were not included in the analysis. Table 2 lists recipient and corresponding donor CYP3A5*3, CYP3A4*1G, *SUMO4*, and *NR112* allele frequencies. These two genotypes showed no deviation from Hardy–Weinberg equilibrium (*p* > 0.05).

**TABLE 1:** Demographic and clinical characteristics of participants after liver transplantation used for model development.

Characteristics	Number or mean ± SD	Median (range)
No. of patients (Male/Female)	176 (150/26)	—
No. of tacrolimus samples	906	—
Age (years)	50.68 ± 9.71	51 (18–74)
Height (m)	1.69 ± 0.06	1.70 (1.54–1.84)
Total body weight, WT (kg)	64.54 ± 11.85	64 (40–104)
Predicted fat free mass, FFM (kg)^a^	50.27 ± 8.50	51.49 (28.87–68.47)
Grafted hepatic weight, HW (g)	1,310.35 ± 207.42	1,300 (603–2,200)
Graft: recipient weight ratio, GRWR (%)	2.14 ± 0.51	2.03 (1.04–3.58)
Hemoglobin, HB (g L^−1^)	106.53 ± 16.69	105 (55–182)
Haematocrit, HCT (%)	31.78 ± 4.98	31.3 (15.4–53.6)
Total serum protein, TP (g L^−1^)	60.4 ± 7.13	60 (38–91)
Serum albumin, ALB (g L^−1^)	37.24 ± 4.05	37 (20–51)
Alanine transaminase, ALT (U L^−1^)	138.99 ± 183.99	69 (4–1765)
Aspartate aminotransferase, AST (U L^−1^)	83.54 ± 143.15	45 (9–2,547)
Alkaline phosphatase, ALP (U L^−1^)	223.7 ± 205.72	156.5 (14–2,125)
γ-Glutamyl transpeptidase, γ-GGT (U L^−1^)	253.91 ± 321.96	148 (11–2,944)
Total serum bilirubin, TBIL (μmol L^−1^)	88.32 ± 90.11	58.25 (5.1–786.3)
Blood uric nitrogen, BUN (mmol L^−1^)	9.65 ± 5.73	8.8 (1.4–53)
Serum creatinine, SCR (μmol L^−1^)	62.15 ± 28.47	57 (19–400)
Creatinine clearance, CLCR (ml min^−1^)^a^	126.83 ± 48.19	124.59 (23.36–380.31)
Methylprednisolone dose (mg day^−1^)	42.31 ± 57.28	20 (0–500)
Postoperative days (day)	11.77 ± 8.88	10 (2–72)
Tacrolimus daily dose (mg day^−1^)	2.73 ± 1.56	2.5 (0.25–8)
Tacrolimus trough concentration (ng ml^−1^)	6.28 ± 3.27	5.96 (0.30–23.11)
Concomitant medication^b^		
Wuzhi capsule	112	—
Characteristics	Number or Mean ± SD	Median (Range)
Calcium channel blocker (Diltiazem/Dihydropyridine)	65 (5/60)	—
Fluconazole	21	—
Voriconazole	35	—
Sulfonylureas	0	—
Glucocorticoid	475	—
PPI (Omeprazole/Pantoprazole)	645 (370/275)	—
Mycophenolate mofetil	53	—

PPI, proton pump inhibitor; SD, standard deviation.

^a^ Calculated from serum creatinine using the Cockcroft-Gault formula: CLCR = [140—age (years)] × weight (kg)/[0.818 × SCR (μmol L^−1^)] × (0.85, if female).

^b^ Data are expressed as number of samples.

**TABLE 2:** Allele frequencies of single nucleotide polymorphisms in CYP3A5*3, CYP3A4, SUMO4, and *NR112* genes of the development dataset.

Single nucleotide polymorphisms	Number of recipients	Frequency (%)
**Recipients**		
CYP3A5*3 (A6986G, rs776746)		
AA (*1/*1)	12	6.82
GA (*1/*3)	71	40.34
GG (*3/*3)	93	52.84
CYP3A4*1G (G20230A, rs2242480)		
GG (*1/*1)	99	56.25
GA (*1/*1G)	61	34.66
AA (*1G/*1G)	16	9.09
SUMO4 (163A>G, rs237025)		
AA	83	47.16
AG	79	44.89
GG	14	7.95
NR112 (8055C>T, rs2276707)		
CC	37	21.02
CT	116	65.91
TT	23	13.07
**Donors**		
CYP3A5*3 (A6986G, rs776746)		
AA (*1/*1)	11	6.25
GA (*1/*3)	84	47.73
GG (*3/*3)	81	46.02
CYP3A4*1G (G20230A, rs2242480)		
GG (*1/*1)	92	52.27
GA (*1/*1G)	75	42.61
AA (*1G/*1G)	9	5.11
SUMO4 (163A>G, rs237025)		
AA	86	48.86
AG	74	42.05
GG	16	9.09
NR112 (8055C>T, rs2276707)		
CC	54	30.68
CT	86	48.86
TT	36	20.45

The allele frequencies are found to be in Hardy-Weinberg equilibrium (*p* > 0.05).

### 3.2 Pharmacokinetic modeling

#### 3.2.1 Theory-based linear compartmental model

##### 3.1.1.1 Base model

In the process of theory-based modeling, a one-compartment model with first-order absorption and elimination was selected as the structural model, and *Ka* was fixed at 4.48 h^−1^. Typical population values of the PK parameters *CL*_*pl*_ */F* and *V*_*pl*_ */F* were 456 and 10,700 L h^−1^, respectively. The inter-subject variabilities of *CL*_*pl*_ */F* and *V*_*pl*_ */F* were 42.2 and 64.4%, respectively. Residual variability was described using a proportional error model. Table 3 present the estimated results.

**TABLE 3:** Population pharmacokinetic parameters of MM and theory-based base models.

Parameters	Estimate	RSE (%)	95%CI	BSV (%)	RUV
**Theory-based model**					
CL_Pl_/F (L h^−1^)	456	3.6	424–488	42.2	14.3%
V_Pl_/F (L)	10700	10.2	8,564–12836	64.4	
*Ka* (h^−1^)	4.48 fixed	—	—	—	
**MM model**					
V_m_ (mg day^−1^)	6.1	6	5.385–6.815	—	0.48 mg day^−1^
K_m_ (ng ml^−1^)	6.19	13.6	4.542–7.838	74.8	29%

CL_Pl_/F, the apparent plasma clearance; V_Pl_/F, the apparent plasma volume of distribution; *Ka*, absorption rate constant; V_m_, the maximum dose rate (daily dose) at the steady state; K_m_, the Michaelis constant which denotes the steady-state trough concentration at half-maximal dose rate; BSV, between subject variability; RUV, unexplained residual error; CI, confidence interval; RSE, relative standard error; MM, Michaelis-Menten.

The base popPK model was described as follows:

$$Ka\left(h^{-1}\right)=4.48$$

$$CL_{Pl}/F\left(L\;h^{-1}\right)=456\times e^{\eta 1}$$

$$V_{Pl}/F\left(L\right)=10,700\times e^{\eta 2}$$

##### 3.1.1.2 Covariate models

The stepwise method was used to examine covariate influence on PK parameters. OFV decreased significantly (∆OFV = −357.745) upon DD inclusion in *CL*_*Pl*_ */F* in the form of a nonlinear power function. Inter-individual *CL*_*Pl*_ */F* variability decreased by 16.8%, which markedly improved the goodness-of-fit of the model. These results further suggested that tacrolimus exhibits nonlinear PK characteristics. We then included POD in *V*_*Pl*_ */F*, and the OFV further decreased by 49.935. AST, tacrolimus DD, co-medication with triazole antifungal drugs (TAF), and the recipient CYP3A5*3 genotype were added to *CL*_*Pl*_ */F* during the forward inclusion process. No covariate was removed during backward elimination. Supplementary Table S1 list the processes of forward inclusion and backward elimination. The final popPK model was described as follows:

$$Ka\left(h^{-1}\right)=4.48$$

$$CL_{Pl}/F\left(L\;h^{-1}\right)=234\times {\left(\text{AST}/45\right)}^{-0.216}\times 3.51\times \text{DD}/\left(2.44+\text{DD}\right)\times 1.168\left(\text{if}\;\text{recipient}\;CYP3A5*1*3\;\text{or}\;CYP3A5*1*1\right)\times 0.425\left(\text{if}\;\text{combined}\;\text{with}\;\text{TAF}\right)V_{Pl}/F\left(L\right)=11000\times e^{0.887\times \left(POD/15\right)}$$

where DD is in mg day^−1^.

All parameter precisions represented by a standard error were acceptable. The population pharmacokinetic parameter estimates and precision of the final model with covariates are presented in Table 4.

**TABLE 4:** Pharmacokinetic parameter estimates for the final models and Bootstrap results.

Parameters	Final model			Bootstrap		Bias (%)
	Estimate	RSE (%)	95%CI	Median	95%CI	
**Theory-based compartmental model**						
*Ka* (h^−1^)	4.48 fixed	—	—	4.48 fixed	—	—
CL_Pl_/F (L h^−1^)	234	45.7	24.28–443.72	231.12	203.96–283.56	−0.23
θ_DDmax_	3.51	55.8	0.332–7.352	3.51	3.09–4.39	0
θ_DD50_	2.44	25	1.244–3.636	2.45	1.43–3.95	1.14
θ_AST_	(−0.216)	19.8	(−0.3)-(-0.312)	(-0.211)	(−0.299)-(−0.139)	−2.31
θ_VRCZ_	(−0.575)	17	(−0.766)-(−0.384)	(−0.571)	(−0.871)-(−0.312)	−0.69
θ_CYP3A5*1_	0.168	37.1	0.046–0.29	0.167	0.056–0.299	−0.59
V_Pl_/F (L)	11000	10.8	8,667.6–13332.4	10822.2	8,696.0–13072.5	−1.62
θ_POD_	0.887	12.3	0.673–1.101	0.886	0.727–1.159	−0.11
Between subject variability						
ω_CLpl/F_ (%)	29.7	8.2	—	29.2	24.5–34.4	−1.68
ω_Vpl/F_ (%)	59.2	11.5	—	59.1	39.3–71.3	−0.17
Residual unexplained error						
δ_1_ (%)	30.8	7.7	—	30.7	28.2–32.9	−0.32
**MM model**						
V_m_ (mg day^−1^)	6.62	5.2	5.952–7.288	6.63	6.03–7.50	0.15
K_m_ (ng ml^−1^)	6.46	14	4.686–8.234	6.45	4.90–8.73	−0.15
θ_POD_	0.277	22.6	0.154–0.4	0.272	0.152–0.404	−1.80
θ_HCT_	1.16	22.8	0.641–1.679	1.14	0.598–1.685	−1.72
θ_TBIL_	0.286	23	0.157–0.415	0.281	0.156–0.429	−1.75
θ_CYP3A5*1_	(-0.365)	19.4	(−0.504)–(−0.226)	(−0.362)	(−0.500)–(−0.210)	−0.82
θ_VRCZ_	2.15	42.3	0.368–3.932	2.13	0.554–4.417	−0.93
Between subject variability						
ω_Km_	65.3%	7.8	—	64.7%	55.3%–74.1%	−0.92
Residual unexplained error						
δ_1_ (%)	20.4	10.5	—	20.2	14.4–24.4	−0.098
δ_2_ (mg day^−1^)	0.568	10.2	—	0.559	0.436–0.699	−1.58

CL_Pl_/F, the apparent plasma clearance; V_Pl_/F, the apparent plasma volume of distribution; *Ka*, absorption rate constant; V_m_, the maximum dose rate (daily dose) at the steady state; K_m_, the Michaelis constant which denotes the steady-state trough concentration at half-maximal dose rate; ω, between subject variability; δ_1_, proportional residual error; δ_2_, additive residual error; θ, the coefficient of the included covariates on the parameters; CI, confidence interval; RSE, relative standard error; CYP3A5*1, CYP3A5*1*3 and CYP3A5*1*1 expresser; MM, Michaelis-Menten; Bias, prediction error.

$Bias\%=\frac{Bootstrap-NONMEM}{NONMEM}\times 100\%$

Where NONMEM, represents the PK, parameters estimates of the final model and Bootstrap represents the PK, parameters median values obtained from the nonparametric bootstrap procedure.

#### 3.2.2 Nonlinear Michaelis-Menten empirical model

##### 3.2.2.1 Base model

To better estimate the parameters, *V*_*m*_ inter-subject variability was fixed at 0. Typical population PK parameter values were 6.1 mg day^−1^ for *V*_*m*_ and 6.19 ng ml^−1^ for *K*_*m*_; *K*_*m*_ inter-individual variability was 74.8%. The combination error model best described the residual variability. Table 3 summarize the parameter estimates.

The base popPK model was described as follows ***:***

$$V_{m}\left(\text{mg}\;{\text{day}}^{-1}\right)=6.1$$

$$K_{m}\left(\text{ng}\;{\text{mL}}^{-1}\right)=6.19$$

##### 3.2.2.2 Covariate models

The MM model describes the nonlinear relationship between drug DD and steady state C_0_. Following POD addition to *K*_*m*_ in the form of an exponential (∆OFV = −36.688) or power function (∆OFV = −90.911), OFV significantly decreased. Upon including the time factors (*10/θ*) into *K*_*m*_ based on parameter and covariate correlation analysis (Supplementary Figure S1), OFV further decreased by 67.657, suggesting that *K*_*m*_ exhibits the characteristics of a time-dependent variant. However, the addition of WT did not yield significant changes (*p* < 0.05).

Because of the predominant hepatic metabolism and bile-based excretion of tacrolimus, we investigated the effects of ALT and TBIL levels as covariates on model fitting. TBIL inclusion in *K*_*m*_ (∆OFV = −13.328) led to approximately 5% decrease in *K*_*m*_ inter-individual variability. Consistent with its association with erythrocyte levels *in vivo* and effects on tacrolimus distribution, HCT incorporation into *K*_*m*_ improved model fitting (△OFV = −38.741). Addition of the CYP3A5*3 binary covariate to *K*_*m*_ also yielded significant change (△OFV = −15.933), whereas including other polymorphisms did not improve model goodness-of-fit, regardless of whether two or three classification covariates were evaluated.

TAFs, such as voriconazole and fluconazole, were investigated as binary variables because they can significantly affect tacrolimus metabolism. Including this covariate in *K*_*m*_ decreased OFV by more than 3.84 (△OFV = −43.629). The addition of other covariates, such as concomitant medication hormones, did not significantly affect the forward inclusion process. The final covariates added in this process were POD, time factors (*10/θ*), TBIL, HCT, CYP3A5*3, and TAF. No covariate was removed during backward elimination. Supplementary Table S2 present the results.

The final popPK model was described as follows:

$$V_{m}\left(\text{mg}\;{\text{day}}^{-1}\right)=6.62$$

$$K_{m}\left(\text{ng}\;{\text{ml}}^{-1}\right)=6.46\times {\left(\text{POD}/15\right)}^{0.277}\times {\left(\text{HCT}/31\right)}^{1.16}\times {\left(\text{TBIL}/58.25\right)}^{0.286}\times 0.635\left(\text{if}\;\text{recipient}\;CYP3A5*1*3\;\text{or}\;CYP3A5*1*1\right)\times 3.15\left(\text{if}\;\text{combined}\;\text{with}\;\text{TAF}\right).$$

All parameter precisions represented by a standard error were acceptable. The population pharmacokinetic parameter estimates and precision of the final model with covariates are listed in Table 4.

### 3.2 Model evaluation

#### 3.2.2 Theory-based linear compartmental model

**FIGURE 1:**
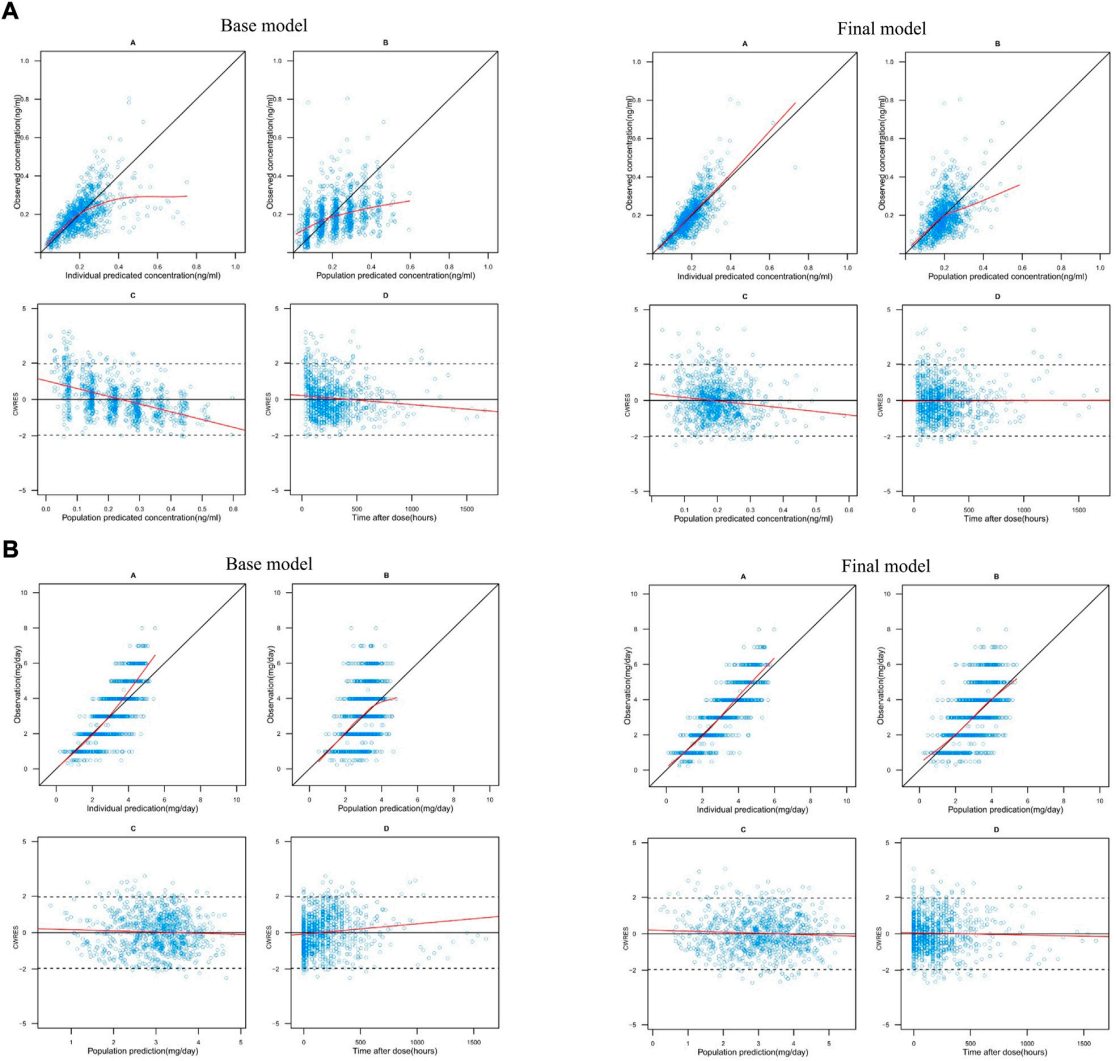
**(A)** Goodness-of-fit plots for the theory-based base and final model. In each model: A. observations (*y*-axis) vs. individual predictions (*x*-axis), B. observations (*y*-axis) vs. population predictions (*x*-axis), C. conditional weighted residuals (CWRES) (*y*-axis) vs. population predictions (*x*-axis) and D. CWRES (*y*-axis) vs. time after dose (*x*-axis). In plots A and B, the black solid lines are identity lines. In plots C and D, the black solid and dashed lines represent the y = 0 and y = ±1.96 reference lines, respectively. The red solid lines represent local polynomial regression (lowess) lines. **(B)** Goodness-of-fit plots for the MM base and final model. In each model: A. observations (*y*-axis) vs. individual predictions (*x*-axis), B. observations (*y*-axis) vs. population predictions (*x*-axis), C. conditional weighted residuals (CWRES) (*y*-axis) vs. population predictions (*x*-axis) and D. CWRES (*y*-axis) vs. time after dose (*x*-axis). In plots A and B, the black solid lines are identity lines. In plots C and D, the black solid and dashed lines represent the y = 0 and y = ±1.96 reference lines, respectively. The red solid lines represent local polynomial regression (lowess) lines.

Figure 1A shows the GOF plots of the base and final models. No significant structural bias or obvious systematic deviations were found in the final model, and data fitting was improved compared with the base model. Most *CWRES* values of the final model were within ±2, indicating an acceptable fit.

The MAPE, *F*_*20*_, *F*_*30*_, MAIPE, *IF*_*20*_, *IF*_*30*_, MDPE, and MDIPE values of the theory-based final model were 28.08, 37.31, 53.2, 17.41, 56.18, 75.5, 5.27, and −1.59%, respectively. Bootstrap analysis was successful in 96.1% of the 1,000 runs. The PK parameters median values and 2.5–97.5% estimates obtained from the bootstrap approximated those obtained with the original dataset with <3% bias, confirming the stability and robustness of the final model (Table 4).

**FIGURE 2:**
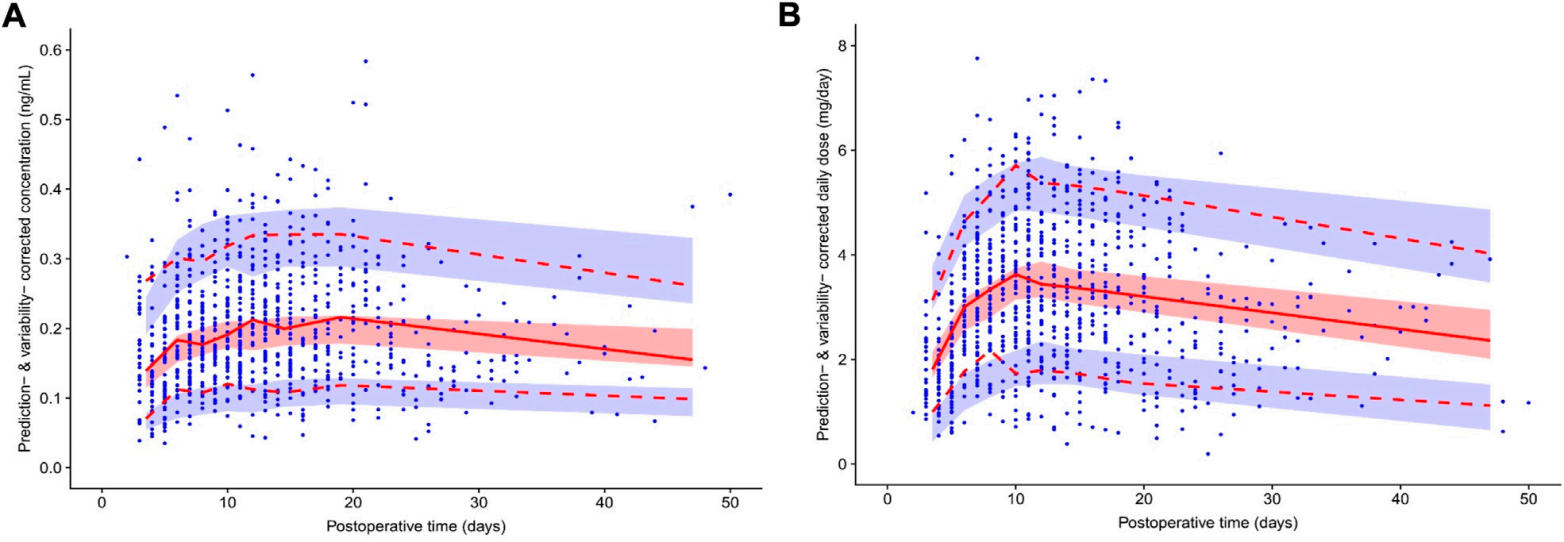
Prediction- and variability-corrected visual predictive check (pvcVPC) plot of the theory-based final model plot **(A)** and final MM model plot **(B)**. The red solid line represents the prediction- and variability-corrected median observed concentration, and the semitransparent red shaded area represents the simulation-based 95% confidence intervals (CIs) for the median. The red dashed lines represent the corrected observed 10th and 90th percentiles, and the semitransparent blue shaded areas represent the simulation-based 95% CIs for the corresponding predicted percentiles from the final model. The blue dots represent the prediction- and variability-corrected observations.

Figure 2A shows the *pvcVPCs* of the final model. The 10th, 50th, and 90th percentiles of the observations during the 50 days after surgery were within the 95% confidence intervals (CIs) of the corresponding prediction percentiles for the final model. Only the 10th and 90th percentiles of the observations on the fourth and sixth day after surgery fell slightly outside the 95% CI, revealing an acceptable agreement between the simulated and observed concentrations at most sampling time points.

**FIGURE 3:**
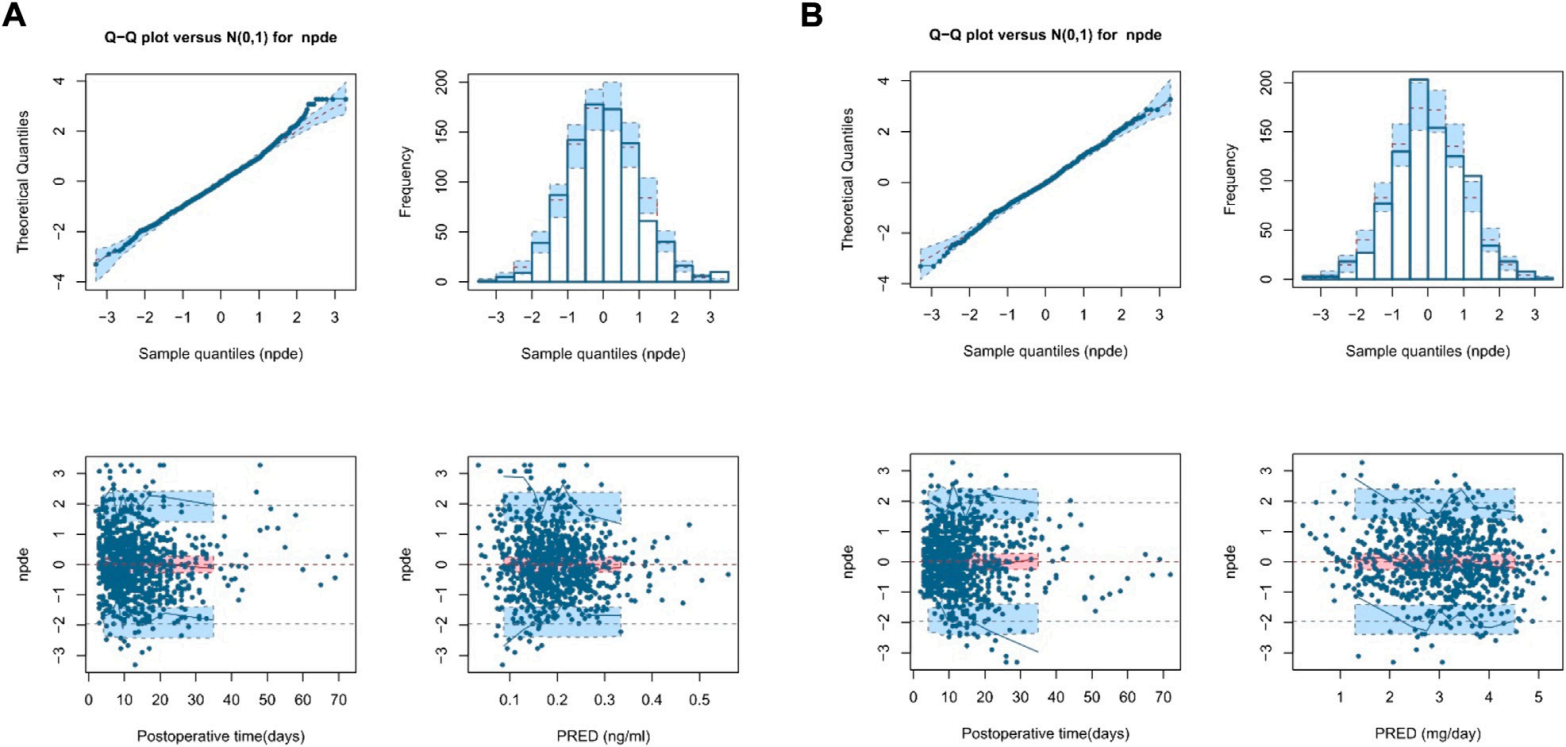
Normalized prediction distribution error (NPDE) plot of the theory-based final model plot **(A)** and MM final model plot **(B)**. The plots are quantile-quantile plot of the distribution of NPDE against the theoretical distribution (semitransparent blue fields) (top left), histogram of the distribution of NPDE against the theoretical distribution (semitransparent blue fields) (top right), scatter plot of NPDE vs. postoperative time (days) (bottom left), and scatter plot of NPDE vs. population predictions (bottom right). In the scatter plots, the red solid lines represent the median NPDE of the observations, and semitransparent red fields represent the simulation-based 95% confidence intervals (CIs) for the median. Blue solid lines represent the NPDE of the observed 5th and 95th percentiles, and semitransparent blue fields represent the simulation-based 95% CIs for the corresponding predicted percentiles from the model. The blue dots represent the NPDE of the observations.

Figure 3A, Supplementary Table S3 show the NPDE results. Quantile–quantile plots and histograms confirmed the normality of NPDE. However, the assumption of a normal distribution for the differences between predictions and observations was statistically unacceptable, with adjusted *p*-values within 0.05 for the global test, indicating the limited predictability of the final model.

#### 3.2.3 Nonlinear Michaelis–Menten empirical model

Figure 1B shows the GOF plots of the base and final models. The final model exhibited no significant structural bias or systematic deviation. The data fitting was improved compared to that of the base model. Most *CWRES* values of the final model were within ±2, indicating an acceptable fit.

**FIGURE 4:**
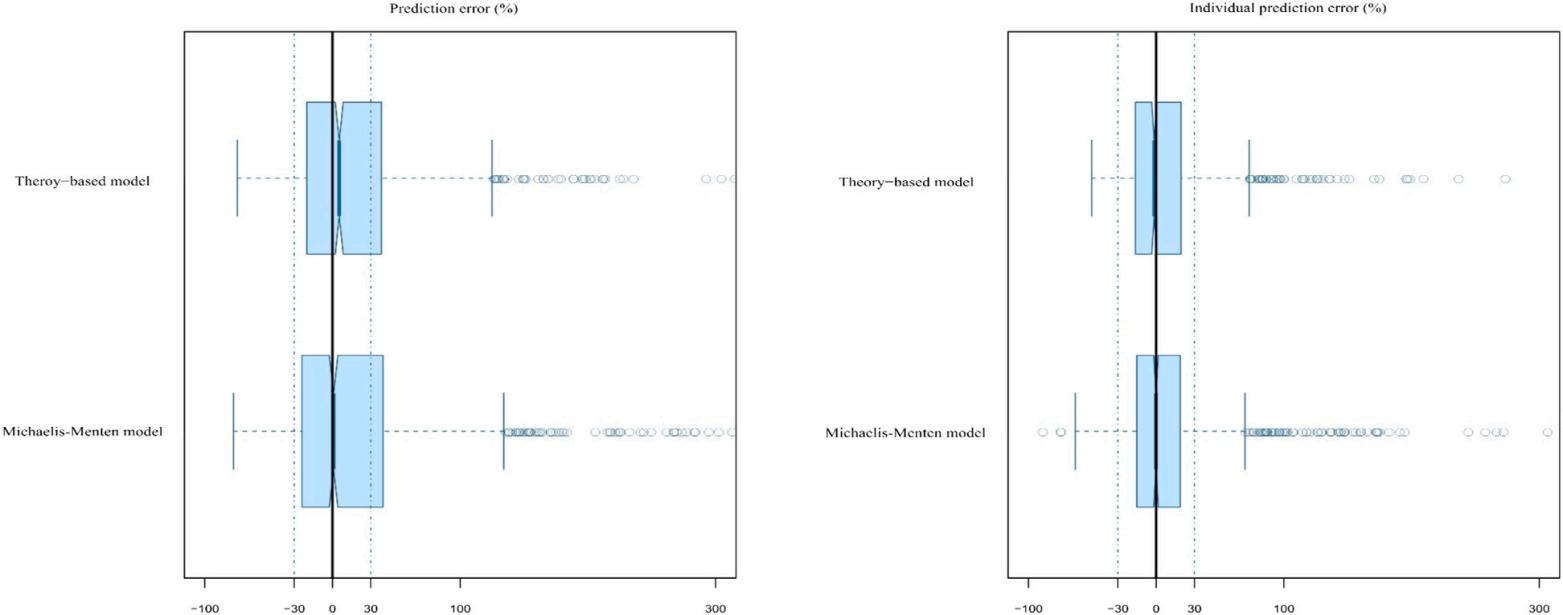
Box plots of the prediction error (PE%) and individual prediction error (IPE%) for the two established final models. Black solid lines and blue dotted lines are reference lines indicating PE% or IPE% of 0% and ±30%, respectively.

The MAPE, *F*_*20*_, *F*_*30*_, MAIPE, *IF*_*20*_, and *IF*_*30*_ of the MM final model were 29.21, 34.11, 51.32, 16.63, 57.95, and 74.06%, respectively. However, its MDPE and MDIPE were 0.84 and −0.11%, respectively, which were both significantly lower than those of the theory-based final model, indicating superior prediction accuracy. The results are presented in Table 5 and Figure 4. Bootstrap analysis was successful in 99.8% of the 1,000 runs. The obtained PK parameters median values and 2.5–97.5% estimates approximated those obtained with the original dataset with <2% bias, confirming the stability and robustness of the final model (Table 4).

**TABLE 5:** The results of prediction error evaluation for the MM and theory-based final models.

Models	PE				IPE			
	MDPE	MAPE	F_20_	F_30_	MDIPE	MAIPE	IF_20_	IF_30_
MM final model	0.84	29.21	34.11	51.32	−0.11	16.63	57.95	74.06
Theory-based final model	5.27	28.08	37.31	53.2	−1.59	17.41	56.18	75.5

MM, Michaelis-Menten; MDPE (%), median prediction error; MAPE (%), median absolute prediction error; F_20_ (%) and F_30_ (%), percentage of prediction error ≤ ±20% and ±30%, respectively. MDIPE (%), median individual prediction error; MAIPE (%), median absolute individual prediction error; IF_20_ (%) and IF_30_ (%), percentage of individual prediction error ≤ ±20% and ±30%, respectively.

Figure 2B shows the *pvcVPCs* of the final model. The 10th, 50th, and 90th percentiles of the observations during the 50 days after surgery were within the 95% CIs of the corresponding prediction percentiles for the final model. However, at around the eighth day after surgery, the 10th percentile of the observed data deviated slightly from the 95% CI of the corresponding percentile of the simulated data, which had a limited impact on the final model overall prediction efficiency.

Figure 3B, Supplementary Table S3 show the *NPDE* results. Quantile–quantile plots and histograms confirmed NPDE normality and good final model predictability. The assumption of a normal distribution for the differences between predictions and observations was acceptable with adjusted *p*-values of 0.25 for the global test, indicating a superior predictive performance compared with that of the theory-based final model.

Compared with that of the base model, the data fitting was markedly improved; no significant structural bias or obvious trends were found in the two final models. The bootstrap and pvcVPC results showed that both models had good robustness and comparable predictive performance. The success rate determined using the MM model bootstrap method was slightly higher than that of the theory-based model. However, unlike the final theory-based model, the MM model showed better performance in the NPDE regarding to the normal distribution and global tests, indicating the superiority of the latter. In addition, the MDPE and MDIPE of the MM final model also implied its superiority and higher reliability.

### 3.3 Model-informed individualized dosing

A Monte Carlo simulation for the starting tacrolimus dose was conducted based on the final established nonlinear MM model, which exhibited superior predictive performance. The steady-state target *C*_*0*_ of tacrolimus should be maintained at 8–12 ng ml^−1^ on the seventh day after liver transplantation in typical adult patients. The HCT was set from 20 to 50% in 10% steps. At each HCT level, TBIL was set to four levels (<17.1, 17.1–85.5, 85.5–171, and >171 μmol L^−1^) according to the severity of jaundice, and the recipients were divided into poor (CYP3A5*3*3) and intermediate metabolizers (CYP3A5*1*3 or CYP3A5*1*1). Supplementary Table S4 presents the specific simulated scenarios.

The required dose was calculated using *V*_*m*_, *K*_*m*_, and the desired target *C*_*0*_. For example, when HCT levels were between 20 and 50%, for CYP3A5*3*3 expressers with and without TAF co-therapy, a starting tacrolimus DD of 1–3.25 and 2–5 mg, respectively, could achieve a target steady-state *C*_*0*_ of 8–12 ng ml^−1^, whereas the initial DD should increase to 1.25–4 and 3.25–5.5 mg for CYP3A5*1*3 or CYP3A5*1*1 carriers, respectively.

**FIGURE 5:**
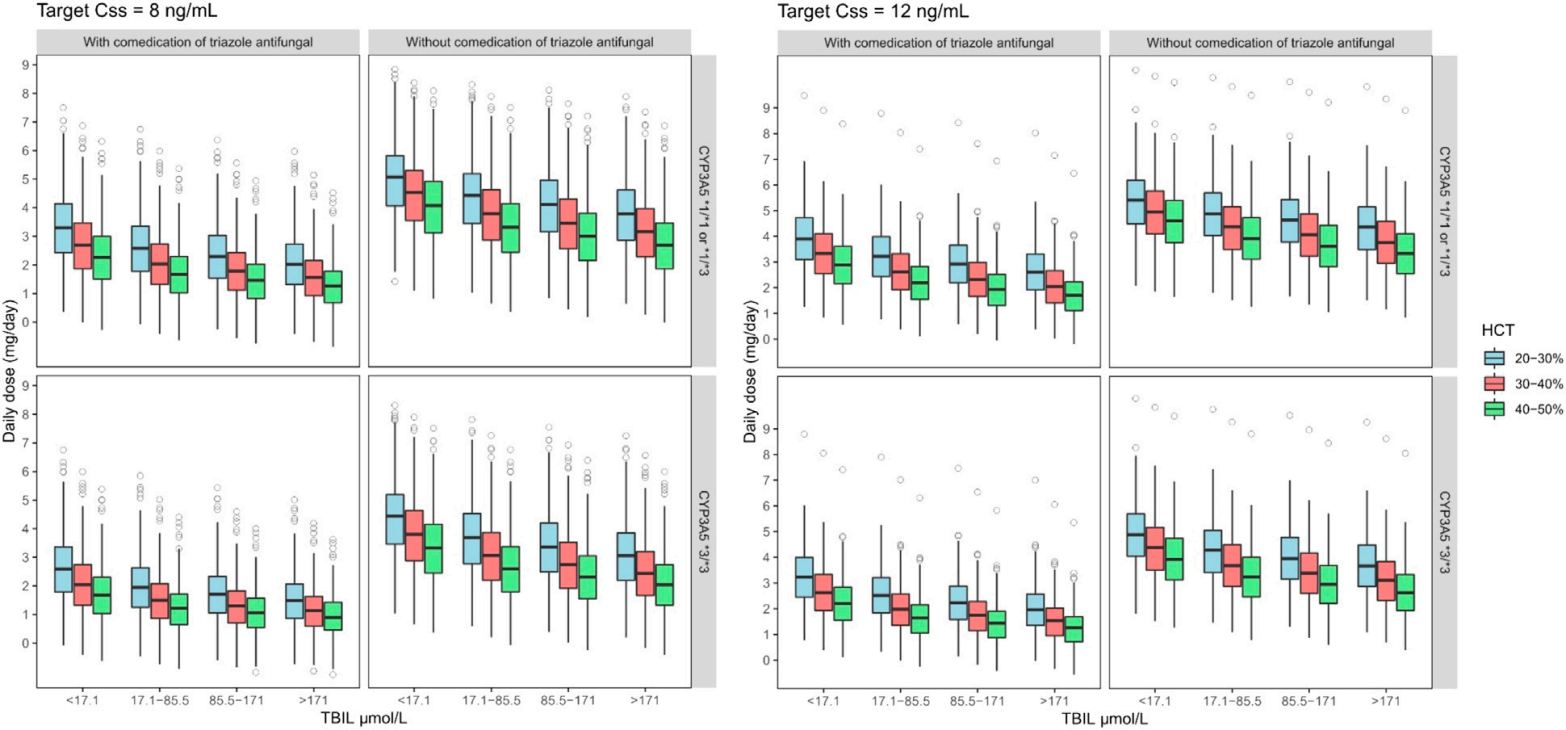
Boxplots of the distributions of simulated tacrolimus daily dose for CYP3A5*1*1 or *1*3 and *3*3 on target steady-state trough concentrations at 8–12 ng ml^−1^ for co-administered with triazole antifungal group in different TBIL and HCT levels. The bold horizontal bars in the middle show the median values, whereas the outer boundaries of the boxes represent the ranges of the 25th and 75th percentiles (interquartile ranges). The whiskers indicate the maximum and the minimum values of daily dose. Dots represent the outliers.

Furthermore, as tacrolimus is mainly excreted through bile, its concentration increases significantly with increased TBIL level, leading to a lower dose requirement. For example, for CYP3A5*1*3 or CYP3A5*1*1 patients with TAF co-therapy and a HCT of 20–30%, the starting tacrolimus DD should be reduced from 3.25 to 4 to 2–2.5 mg when TBIL increased from <17.1 to >171 μmol L^−1^ Figure 5 and Table 6 show the simulation results.

**TABLE 6:** MM model-informed individualized initial dose regimens based on simulation.

CYP3A5 genotype	With TAF co-therapy			Without TAF co-therapy		
	HCT 20%–30%	HCT 30%–40%	HCT 40%–50%	HCT 20%–30%	HCT 30%–40%	HCT 40%–50%
CYP3A5*1/*1 or CYP3A5*1/*3						
TBIL<17.1 μmol L^−1^	3.25–4 mg	2.75–3.25 mg	2.25–3 mg	5–5.5 mg	4.5–5 mg	4–4.5 mg
17.1 ≤ TBIL<85.5 μmol L^−1^	2.5–3.25 mg	2–2.75 mg	1.75–2.25 mg	4.5–5 mg	3.75–4.5 mg	3.25–4 mg
85.5 ≤ TBIL<171 μmol L^−1^	2.25–3 mg	1.75–2.25 mg	1.5–2 mg	4–4.5 mg	3.5–4 mg	3–3.5 mg
TBIL≥171 μmol L^−1^	2–2.5 mg	1.5–2 mg	1.25–1.75 mg	3.75–4.25 mg	3.25–3.75 mg	2.75–3.25 mg
CYP3A5*3/*3						
TBIL<17.1 μmol L^−1^	2.5–3.25 mg	2–2.5 mg	1.75–2.25 mg	4.5–5 mg	3.75–4.5 mg	3.25–4 mg
17.1 ≤ TBIL<85.5 μmol L^−1^	2–2.5 mg	1.5–2 mg	1.25–1.75 mg	3.75–4.25 mg	3–3.75 mg	2.5–3.25 mg
85.5 ≤ TBIL<171 μmol L^−1^	1.75–2.25 mg	1.25–1.75 mg	1–1.5 mg	3.25–4.25 mg	2.75–3.75 mg	2.25–3.25 mg
TBIL≥171 μmol L^−1^	1.5–2 mg	1.25–1.5 mg	1–1.25 mg	3–3.75 mg	2.5–3 mg	2–2.5 mg

## 4 Discussion

Tacrolimus is a well-known drug exhibiting linear kinetics in most previous studies (Staatz et al., 2003; Li et al., 2007; Zhu et al., 2015; Ji et al., 2018). However, we found that it follows nonlinear kinetics in patients with kidney transplantation and nephrotic syndrome (Zhao et al., 2016; Huang et al., 2020). In this study, we further confirmed tacrolimus nonlinearity in adult patient populations undergoing liver transplantation using a large sample size. To further address whether tacrolimus PK changes with oral *DD* rate and explore the potential mechanisms of the identified nonlinear kinetics, the nonlinear behavior of tacrolimus PK in adult liver transplant recipients was modeled and compared with that from the theory-based linear model established by the same independent dataset prospectively collected from Huashan Hospital.

The MM model, an efficient population approach to describe the nonlinear correlation between the dose and steady-state *C*_*0*_, was employed as it can describe the overall nonlinear behavior across the entire PK process. Based on the superior predictability in adult liver transplant recipients, the MM model might constitute a promising approach for tacrolimus starting dose determination, implying that the target *C*_*0*_ range can be more easily reached and lead to more effective clinical outcomes in practical application. In the MM final model, the typical value of *V*_*m*_ was 6.62 mg day^−1^ (95% CI, 5.952–7.288), indicating that a tacrolimus DD exceeding 6.62 mg may lead to drug accumulation and adverse reactions. The typical *K*_*m*_ was 6.46 ng ml^−1^ (95%CI, 4.686–8.234), indicating that for a steady-state concentration <6.46 ng ml^−1^, the drug exhibited saturated erythrocyte binding. Thus, its nonlinearity would no longer influence dose rate adjustment when the steady-state *C*_*0*_ was ≤6.46 ng ml^−1^.

However, tacrolimus nonlinearity in adult liver transplant recipients may differ from that in patients with renal transplantation or PNS. For example, the values of *V*_*m*_ and *K*_*m*_ in adult liver transplant recipients were significantly higher than those in patients with PNS (*V*_*m*_: 1.92 mg kg^−1^; *K*_*m*_: 1.98 ng ml^−1^) (Cai et al., 2020) and adult renal transplant recipients (*V*_*m*_: 5.54 mg day^−1^; *K*_*m*_: 2.36 ng ml^−1^). The discrepancy between these two populations might be attributed to various changes during childhood growth (Yokoi, 2009), lower total DD, and lower target tacrolimus *C*_*0*_ requirement (5–10 ng ml^−1^) (Subspecialty Group of Nephrology S O P C M A, 2010; Vasudevan et al., 2021) than in adult liver transplant recipients (8–12 ng ml^−1^ within 3 months after transplantation). Poor hepatic function might also significantly affect first-pass tacrolimus metabolism in liver transplant recipients; these also receive different steroid dosages (Bekersky et al., 2001; Shimomura et al., 2002; Vanhove et al., 2016). Additionally, most liver transplant recipients included in our study were hepatitis B-, hepatitis C-, or hepatitis E-positive, unlike most PNS or renal transplant recipients. Hepatitis virus replication in hepatocytes alters the CYP3A system, leading to reduced tacrolimus metabolism (Horina et al., 1993).

Factors contributing to tacrolimus nonlinearity may involve specific drug properties including poor water solubility (1–2 μg ml^−1^), high absorption variability, and acceleration of metabolism at high doses (Lee et al., 2006). Furthermore, post-transplantation gastrointestinal dysfunction, changes in metabolizing enzymes and P-gp activity with POD, and gradually decreasing hepatic and intestinal CYP3A and P-gp induction by tapered co-administered steroid dosing cause higher fluctuations in absorption and metabolism leading to more pronounced nonlinear PK behavior (Chow et al., 1997; Christians et al., 2002; Lee et al., 2006; Tubic et al., 2006).

Nonlinear tacrolimus PK may also result from poor hepatic function and low HCT levels, as patients with liver transplantation usually suffer from gastrointestinal bleeding and postoperative bone marrow suppression early after surgery. Descending plasma protein caused by poor hepatic function, in addition to low HCT levels, could lead to saturated tacrolimus concentration-dependent binding to albumin and erythrocytes, consequently increasing the free tacrolimus proportion (Chow et al., 1997). Free tacrolimus can pass through the cell membrane, undergo CYP3A-mediated metabolism, and be cleared by the liver and kidney (Jusko et al., 1995), leading to nonlinear distribution and elimination. The relationship between nonlinear PK and distribution and elimination *in vivo* could be explained by the present results, as HCT constituted a significant variable in the developed MM model, and saturated tacrolimus–erythrocyte binding was incorporated into the theory-based final model.

Notably, early classic PK studies in healthy individuals showed that the tacrolimus PK at a single oral dose of 3, 7, and 10 mg indicated a linear process, and the clearance rate was not related to the dose (Bekersky et al., 1999). Several factors may underlie these discrepancies. 1) The median tacrolimus DD in adult liver transplant recipients enrolled in the present study was 2.5 mg; i.e., the DD in 50% of patients was <3 mg, which was not within the dose range of 3–10 mg. 2) In contrast to the steady-state levels in a healthy population, HCT and CYP3A activity in the intestine and liver gradually recovered with POD after surgery (Brooks et al., 2016), further contribute to the nonlinear PK characteristics in adult liver transplant recipients.

The superior predictive performance of the nonlinear MM empirical model suggests that this model provides a novel perspective for future investigations, and that saturated binding to erythrocytes only partially explains the nonlinear tacrolimus PK. Nevertheless, considering that only *C*_*0*_ values were available, the function of the DD might reflect nonlinearity in the clearance process. Further studies are needed to confirm the sources of tacrolimus PK nonlinearity and investigate the physiological significance of *K*_*m*_ and *V*_*m*_.

Our study shows that in theory-based modeling, PK parameters were estimated based on *C*_*p*_ (Hebert et al., 2013; Storset et al., 2014b). For CYP3A5*3*3 patients with an AST of 45 U L^−1^, administered TAF-free treatment and 1 mg tacrolimus DD, estimated *CL*_*pl*_ */F* was 234 L h^−1^, which is much lower than the reported value (473–695 L h^−1^) for non-Asian liver transplant (Jusko et al., 1995; Sam et al., 2006) and kidney transplant populations (811 L h^−1^) (Storset et al., 2014a). This discrepancy cou1d be attributed to the different races and to lower tacrolimus bioavailability in recipients of renal *versus* liver transplants (Staatz and Tett, 2004).

Both final models indicated that only the CYP3A5*3 genotype recipients significantly affected tacrolimus PK in liver transplant patients, consistent with published data (Zhu et al., 2015; Teng et al., 2022). Liver transplant recipients with CYP3A5*1*3 or CYP3A5*1*1 had 16.8% higher *CL*_*pl*_ */F* and 36.5% lower *K*_*m*_ values than those of CYP3A5*3*3 carriers. Other tested genetic polymorphisms, including CYP3A4*1G (rs2242480)*, SUMO4* (rs237025), and *NR112* (rs2276707) genotypes of both donors and recipients did not improve the predictive performance of the final models and were not included therein.

Co-therapeutic agents also contributed to tacrolimus PK variability in liver transplant recipients. Our results showed that *CL*_*pl*_ */F* decreased by 57.5% and *K*_*m*_ increased 3.15-fold in liver transplant patients co-administered TAFs such as voriconazole and fluconazole. These agents, commonly used to prevent and treat fungal infection, could reduce tacrolimus metabolism in the jejunum, improve intestinal absorption, and increase bioavailability, leading to higher tacrolimus concentrations by suppressing CYP450 system activity (Albengres et al., 1998; Iwamoto et al., 2015; Mimura et al., 2019). Similarly, calcium channel blockers and Wuzhi capsule, extensively used as a tacrolimus-sparing agent (Li et al., 2011), impact tacrolimus by affecting CYP3A enzyme activity (Jones and MORRIS, 2002; Qin et al., 2013; Zhang et al., 2019), but they failed to be covariates in the final models. Steroids act as CYP3A substrates and inducers (Lunde et al., 2014). Tacrolimus apparent clearance increased up to 1.6-fold with steroid doses >25 mg (Antignac et al., 2007). However, steroids had no significant effect in our study, probably owing to the low dosages (17.27 ± 15.85 mg day^−1^ presented as prednisone).

POD, considered a major surrogate for many time-dependent variables (Pou et al., 1998; Teng et al., 2022), was also identified as an alternative indicator of time-dependent factors in *CL*_*pl*_ */F* and *K*_*m*_ in our study. This may be attributed to progressive CYP3A enzyme activity recovery in the intestine and liver, and steroid dose tapering after transplantation. Other factors and possible mechanisms related to POD effects on tacrolimus PK remain to be determined.

Ultimately, we used the superior MM final model to predict the tacrolimus DD and test the possible clinical impact of the CYP3A5*3 genotype, concomitant medication with TAFs, and HCT levels. The simulation results indicated that a starting DD of 1–3.25 mg for CYP3A5*3*3 patients with TAF co-therapy could reach the target treatment concentration range (8–12 ng ml^−1^) 1 week after surgery, whereas 3.25–5.5 mg was required for CYP3A5*1*3 or CYP3A5*1*1 carriers receiving TAF-free treatment.

This study had some limitations. First, no intensive sampling was available. Only clearance and its covariates were reliably estimated. *C*_*0*_ values alone cannot yield reliable estimates of *V*_*d*_, or adequately illustrate the exact nonlinear PK mechanisms in other processes, especially absorption.

## 5 Conclusion

Tacrolimus PK in recipients of liver transplantation were first compared between theory-based linear compartment and nonlinear MM models through prospective population analysis, and factors contributing to individual PK variability were identified. The MM model, a nonlinear empirical model, better described tacrolimus PK behavior and yielded superior predictive performance based on the large sample size, further confirming tacrolimus nonlinearity. Saturated concentration-dependent erythrocyte binding and the influence of tacrolimus DD on metabolism could partially explain tacrolimus nonlinearity. Notably, tacrolimus nonlinearity in liver transplant patients differed from that in renal transplantation and PNS patients. POD, HCT, TBIL, recipient CYP3A5*3 genotype, and TAF co-therapy represented significant factors in the final MM model, and dosing regimens were proposed. TDM should be strengthened to ensure tacrolimus safety and therapeutic effect, and further efforts should be directed to investigate specific nonlinearities.

## Data Availability

The datasets presented in this study can be found in online repositories. The names of the repository/repositories and accession number(s) can be found in the article/Supplementary Material.
