# Supplementary material for: Prospective population pharmacokinetic study of tacrolimus in adult recipients early after liver transplantation: A comparison of Michaelis-Menten and theory-based pharmacokinetic models
Source: Front Pharmacol. 2022 Nov 9;13:1031969. doi: 10.3389/fphar.2022.1031969 (PMC9681907; doi:10.3389/fphar.2022.1031969)
Supplement: Supplementary file 1 [file DataSheet1.docx]

# *Supporting information*

Additional Supporting Information may be found in the online version of this article at the publisher’s web-site.

## Appendix S1

Genotyping of *CYP3A5*3*, *CYP3A4*1G*, *SUMO4*, and *NR112* single-nucleotide polymorphisms

# *Supplementary tables*

## Table S1

Results of forward inclusion and backward elimination process of the theory-based model

## Table S2

Results of forward inclusion and backward elimination process of the MM model

## Table S3

Results of forward inclusion and backward elimination process of the MM model

## Table S4

Monte Carlo simulation scheme based on MM model in specific simulated scenarios

# *Supplementary figures*

## Figure S1

The correlations between the Km and POD in the MM model

## Appendix S1. Genotyping of CYP3A5*3, CYP3A4*1G, SUMO4, and NR112 single-nucleotide polymorphisms.

DNA was extracted from the whole blood of both liver transplant receipients and their corresponding donors using the TIANamp Blood DNA Kit (Tiangen Biotech Co. Ltd, Beijing, China). The polymerase chain reaction (PCR) was applied to amplify the variant alleles using ABI Veriti 96-Well PCR (Applied Biosystems, Foster City, CA, USA). The volume of amplification reaction was 20 μl, containing 10 μl 2×Taq Master Mix, 7 μl of distilled water, 1 μl of each primer (10 pmol μl^-1^), and 1 μl DNA template (20 ng μl^-1^). The sequences of forward (F) and reverse (R) primers were listed below.

**The sequences of forward (F) and reverse (R) primers for the CYP3A5 genotyping SNPs**

| **Gene** | **Primer** |
| --- | --- |
| *CYP3A5*3*  (rs776746) | F: 5’ CATTTAGTCCTTGTGAGCACTTGAT 3’ |
|  | R: 5’ TAGCACTGTTCTGATCACGTCG 3’ |
| *CYP3A4*1G*  (rs2242480) | F: 5’ AAATGCTTATTGTTAGATGCCACT 3’ |
|  | R: 5’ GGGATTTGAGGGCTTCACTTA 3’ |
| *SUMO4*  (rs237025) | F: 5’ CACAGAAGAAGTCAAGACTGAGAAC 3’ |
|  | R: 5’ CTCCCGTAGGCTGTTGAAAC 3’ |
| *NR112*  (rs2276707) | F: 5’ AGTTATGGGAGGAAGGGAGCTA 3’ |
|  | R: 5’ CCTGCATCAGCACATACTCCT 3’ |

The PCR conditions were as follows: a denaturation at 95 ^o^C for 3 minutes, then 35 cycles of denaturation at 94 ^o^C for 30 seconds, annealing at 55 ^o^C for 25 seconds, and elongation at 72 ^o^C for 30 seconds, followed by a final extension at 72 ^o^C for 5 minutes. The amplified DNA was purified and genotypes were determined by direct sequencing using ABI PRISM 3730XL Sequence Detection System (Applied Biosystems, Foster City, CA, USA). Tm, melting temperature.

# *Supplementary tables*

**Table S1 Forward inclusion and backward elimination process of the theory-based model**

| **Model number** | **Model description** | **OFV** | **ΔOFV** | **Significant** |
| --- | --- | --- | --- | --- |
| **1** | **Base model** | -3397.318 |  |  |
|  | **Forward inclusion** |  |  | (df=1,*P*=0.05,3.84) |
| **2** | ADD FFM on CL_Pl_/F in model 1 | -3370.1 | 27.218 | NO |
| **3** | ADD POD on CL_Pl_/F in model 1 | -3456.323 | -59.005 | YES |
| **3** | ADD DD on CL_Pl_/F in model 1 | -3755.871 | -358.553 | YES |
| **4** | ADD ALT on CL_Pl_/F in model 1 | -3463.12 | -65.802 | YES |
| **5** | ADD AST on CL_Pl_/F in model 1 | -3490.869 | -93.551 | YES |
| **6** | ADD TBIL on CL_Pl_/F in model 1 | -3442.764 | -45.446 | YES |
| **7** | ADD ALB on CL_Pl_/F in model 1 | -3400.463 | -3.145 | NO |
| **8** | ADD CYPR3A5 on CL_Pl_/F in model 1 | -3409.275 | -11.957 | YES |
| **9** | ADD CYPD3A5 on CL_Pl_/F in model 1 | -3400.56 | -3.242 | NO |
| **10** | ADD CYPR3A4 on CL_Pl_/F in model 1 | -3400.588 | -13.27 | NO |
| **11** | ADD CYPD3A4 on CL_Pl_/F in model 1 | -3398.172 | -0.854 | NO |
| **12** | ADD SUMO4R on CL_Pl_/F in model 1 | -3396.994 | 0.324 | NO |
| **13** | ADD SUMO4D on CL_Pl_/F in model 1 | -3396.73 | 0.588 | NO |
| **14** | ADD NR112R on CL_Pl_/F in model 1 | -3397.2 | 0.118 | NO |
| **15** | ADD NR112D on CL_Pl_/F in model 1 | -3397.083 | 0.235 | NO |
| **16** | ADD ALB on CL_Pl_/F in model 1 | 1213.359 | -0.46 | NO |
| **17** | ADD co-voriconazole on CL_Pl_/F in model 1 | -3466.764 | -69.446 | YES |
| **18** | ADD co-wuzhi on CL_Pl_/F in model 1 | -3397.839 | -0.521 | NO |
| **19** | ADD POD on V_Pl_/F in model 1 | -3531.709 | -134.391 | YES |
| **20** | ADD POD on V_Pl_/F in model 3 | -3804.998 | -49.935 | YES |
| **21** | ADD AST on CL_Pl_/F in model 20 | -3857.62 | -52.622 | YES |
| **22** | ADD co-voriconazole on CL_Pl_/F in model 21 | -3899.469 | -41.849 | YES |
| **23** | ADD CYPR3A5 on CL_Pl_/F in model 22 | -3909.751 | -10.282 | YES |
|  | **Backward elimination** |  |  | (df=1,*P*=0.01,6.63) |
| **24** | Remove AST on CL_Pl_/F from model 23 | -3839.723 | 70.028 | YES |
| **25** | Remove CYP3A5 on CL_Pl_/F from model 23 | -3900.889 | 8.862 | YES |
| **26** | Remove DD on CL_Pl_/F from model 23 | -3722.908 | 186.843 | YES |
| **27** | Remove co-voriconazole on CL_Pl_/F from model 23 | -3870.913 | 38.838 | YES |
| **28** | Remove POD on V_Pl_/F from model 23 | -3854.832 | 54.919 | YES |
|  | **Final model** | -3909.751 |  |  |

**Table S2 Forward inclusion and backward elimination process of the MM model**

| **Model number** | **Model description** | **OFV** | **ΔOFV** | **Significant** |
| --- | --- | --- | --- | --- |
| **1** | **Base model** | 1213.819 |  |  |
|  | **Forward inclusion** |  |  | (df=1,*P*=0.05,3.84) |
| **2** | ADD FFM on K_m_ in model 1 | 1213.092 | -0.727 | NO |
| **3** | ADD POD on K_m_ in model 1 | 1122.908 | -90.911 | YES |
| **3** | ADD HCT on K_m_ in model 1 | 1153.329 | -60.49 | YES |
| **4** | ADD CYPR3A5 on K_m_ in model 1 | 1196.74 | -17.079 | YES |
| **5** | ADD CYPR3A4 on K_m_ in model 1 | 1210.231 | -3.588 | NO |
| **6** | ADD CYPD3A5 on K_m_ in model 1 | 1211.83 | -1.989 | NO |
| **7** | ADD CYPD3A4 on K_m_ in model 1 | 1213.258 | -0.561 | NO |
| **8** | ADD SUMO4R on K_m_ in model 1 | 1213.815 | -0.004 | NO |
| **9** | ADD SUMO4D on K_m_ in model 1 | 1212.351 | -1.468 | NO |
| **10** | ADD NR112R on K_m_ in model 1 | 1212.63 | -1.189 | NO |
| **11** | ADD NR112D on K_m_ in model 1 | 1212.983 | -0.836 | NO |
| **12** | ADD AST on K_m_ in model 1 | 1174.578 | -39.241 | YES |
| **13** | ADD TBIL on K_m_ in model 1 | 1145.775 | -68.044 | YES |
| **14** | ADD ALB on K_m_ in model 1 | 1213.359 | -0.46 | NO |
| **15** | ADD co-voriconazole on K_m_ in model 1 | 1174.616 | -39.203 | YES |
| **16** | ADD co-wuzhi on K_m_ in model 1 | 1214.767 | 0.948 | NO |
| **17** | ADD co-voriconazole on K_m_ in model 3 | 1079.279 | -43.629 | YES |
| **18** | ADD HCT on K_m_ in model 17 | 1040.538 | -38.741 | YES |
| **19** | ADD TBIL on K_m_ in model 18 | 1027.21 | -13.328 | YES |
| **20** | ADD ALT on K_m_ in model 19 | 1026.511 | -0.699 | NO |
| **21** | ADD CYPR3A5 on K_m_ in model 19 | 1011.277 | -15.933 | YES |
| **22** | ADD A/θ on K_m_ in model 21 | 943.62 | -67.657 | YES |
| **23** | ADD co-steroid on K_m_ in model 22 | 942.888 | 0.4 | NO |
|  | **Backward elimination** |  |  | (df=1,*P*=0.01,6.63) |
| **24** | Remove A/θ on K_m_ from model 22 | 1011.277 | -67.657 | YES |
| **25** | Remove CYP3A5 on K_m_ from model 22 | 960.319 | 16.699 | YES |
| **26** | Remove TBIL on K_m_ from model 22 | 982.602 | 38.982 | YES |
| **27** | Remove HCT on K_m_ from model 22 | 980.939 | 37.319 | YES |
| **28** | Remove co-voriconazole on K_m_ from model 22 | 1007.695 | 64.075 | YES |
| **29** | Remove POD on K_m_ from model 22 | 974.383 | 30.763 | YES |
|  | **Final model** | 943.62 |  |  |

**Table S3 The NPDE results of MM and theory-based final models**

| **Models** | **Mean (SE)** | **Variance(SE)** | **Skewness** | **Kurtosis** | **P Value** | | | |
| --- | --- | --- | --- | --- | --- | --- | --- | --- |
|  |  |  |  |  | **Wilcoxon^a^** | **Fisher^b^** | **Shapiro-Wilks^c^** | **Global test^d^** |
| Theory-based model | 0.01919(0.034) | 1.066(0.05) | 0.3101 | 0.472 | 0.576 | 0.162 | 0.0000*** | 0.000185*** |
| MM final model | 0.04179(0.033) | 1.017(0.048) | 0.007606 | 0.2243 | 0.212 | 0.715 | 0.0833 | 0.25 |

MM, Michaelis-Menten; SE, standard error.

Signif. codes: '***' 0.001; '**' 0.01; '*' 0.05; '.' 0.1.

a Wilcoxon signed rank test, to test whether the mean is significantly different from 0

b Fisher test for variance, to test whether the variance is significantly different from 1

c Shapiro-Wilks test, to test whether the distribution is significantly different from a normal distribution.

d Global test takes the above 3 tests together with a Bonferroni correction. The p-value for this global test is reported as the minimum of the 3 p-values multiplied by 3, the number of simultaneous tests (or 1 if this value is larger than 1)

**Table S4 Monte Carlo simulation scheme based on MM model in specific simulated scenarios**

| **Schemes** | | **CYP3A5 genotype** | | **HCT**  **（%）** | | | **TBIL**  **（μmol/L）** | | | **TAF co-therapy**  **（0：without；1：with）** | | |
| --- | --- | --- | --- | --- | --- | --- | --- | --- | --- | --- | --- | --- |
| 1 | | ***1/*1 or *1/*3** | | **20-30** | | | ＜17.1 | | | 1 | | |
| 2 | |  | | **30-40** | | | ＜17.1 | | | 1 | | |
| 3 | |  | | **40-50** | | | ＜17.1 | | | 1 | | |
| 4 | | ***3/*3** | | **20-30** | | | ＜17.1 | | | 1 | | |
| 5 | |  | | **30-40** | | | ＜17.1 | | | 1 | | |
| 6 | |  | | **40-50** | | | ＜17.1 | | | 1 | | |
| 7 | | ***1/*1 or *1/*3** | | **20-30** | | | 17.1-85.5 | | | 1 | | |
| 8 | |  | | **30-40** | | | 17.1-85.5 | | | 1 | | |
| 9 | |  | | **40-50** | | | 17.1-85.5 | | | 1 | | |
| 10 | | ***3/*3** | | **20-30** | | | 17.1-85.5 | | | 1 | | |
| 11 | |  | | **30-40** | | | 17.1-85.5 | | | 1 | | |
| 12 | |  | | **40-50** | | | 17.1-85.5 | | | 1 | | |
| 13 | | ***1/*1 or *1/*3** | | **20-30** | | | 85.5-171 | | | 1 | | |
| 14 | |  | | **30-40** | | | 85.5-171 | | | 1 | | |
| *(continues)* | | | | | | | | | | | | |
| **Table S4 continued** | | |  | |  | | |  | | |  | |
| **Schemes** | | | **CYP3A5 genotype** | | **HCT**  **（%）** | | | **TBIL**  **（μmol/L）** | | | **TAF co-therapy**  **（0：without；1：with）** | |
| 15 | |  | | **40-50** | | | 85.5-171 | | | 1 | | |
| 16 | | ***3/*3** | | **20-30** | | | 85.5-171 | | | 1 | | |
| 17 | |  | | **30-40** | | | 85.5-171 | | | 1 | | |
| 18 | |  | | **40-50** | | | 85.5-171 | | | 1 | | |
| 19 | | ***1/*1 or *1/*3** | | **20-30** | | | ≥171 | | | 1 | | |
| 20 | |  | | **30-40** | | | ≥171 | | | 1 | | |
| 21 | |  | | **40-50** | | | ≥171 | | | 1 | | |
| 22 | | ***3/*3** | | **20-30** | | | ≥171 | | | 1 | | |
| 23 | |  | | **30-40** | | | ≥171 | | | 1 | | |
| 24 | |  | | **40-50** | | | ≥171 | | | 1 | | |
| 25 | | ***1/*1 or *1/*3** | | **20-30** | | | ＜17.1 | | | 0 | | |
| 26 | |  | | **30-40** | | | ＜17.1 | | | 0 | | |
| 27 | |  | | **40-50** | | | ＜17.1 | | | 0 | | |
| 28 | | ***3/*3** | | **20-30** | | | ＜17.1 | | | 0 | | |
| 29 | |  | | **30-40** | | | ＜17.1 | | | 0 | | |
| *(continues)* | | | | | | | | | | | | |
| **Table S4 continued** | | | | | | | | | | | | |
| **Schemes** | **CYP3A5 genotype** | | | | | **HCT**  **（%）** | | | **TBIL**  **（μmol/L）** | | | **TAF co-therapy**  **（0：without；1：with）** |
| 30 | |  | | **40-50** | | | ＜17.1 | | | 0 | | |
| 31 | | ***1/*1 or *1/*3** | | **20-30** | | | 17.1-85.5 | | | 0 | | |
| 32 | |  | | **30-40** | | | 17.1-85.5 | | | 0 | | |
| 33 | |  | | **40-50** | | | 17.1-85.5 | | | 0 | | |
| 34 | | ***3/*3** | | **20-30** | | | 17.1-85.5 | | | 0 | | |
| 35 | |  | | **30-40** | | | 17.1-85.5 | | | 0 | | |
| 36 | |  | | **40-50** | | | 17.1-85.5 | | | 0 | | |
| 37 | | ***1/*1 or *1/*3** | | **20-30** | | | 85.5-171 | | | 0 | | |
| 38 | |  | | **30-40** | | | 85.5-171 | | | 0 | | |
| 39 | |  | | **40-50** | | | 85.5-171 | | | 0 | | |
| 40 | | ***3/*3** | | **20-30** | | | 85.5-171 | | | 0 | | |
| 41 | |  | | **30-40** | | | 85.5-171 | | | 0 | | |
| 42 | |  | | **40-50** | | | 85.5-171 | | | 0 | | |
| 43 | | ***1/*1 or *1/*3** | | **20-30** | | | ≥171 | | | 0 | | |
| 44 | |  | | **30-40** | | | ≥171 | | | 0 | | |
| 45 | |  | | **40-50** | | | ≥171 | | | 0 | | |
| *(continues)* | | | | | | | | | | | | |
| **Table S4 continued** | |  | |  | | |  | | |  | | |
| **Schemes** | | **CYP3A5 genotype** | | **HCT**  **（%）** | | | **TBIL**  **（μmol/L）** | | | **TAF co-therapy**  **（0：without；1：with）** | | |
| 46 | | ***3/*3** | | **20-30** | | | ≥171 | | | 0 | | |
| 47 | |  | | **30-40** | | | ≥171 | | | 0 | | |
| 48 | |  | | **40-50** | | | ≥171 | | | 0 | | |

# *Supplementary Figures*


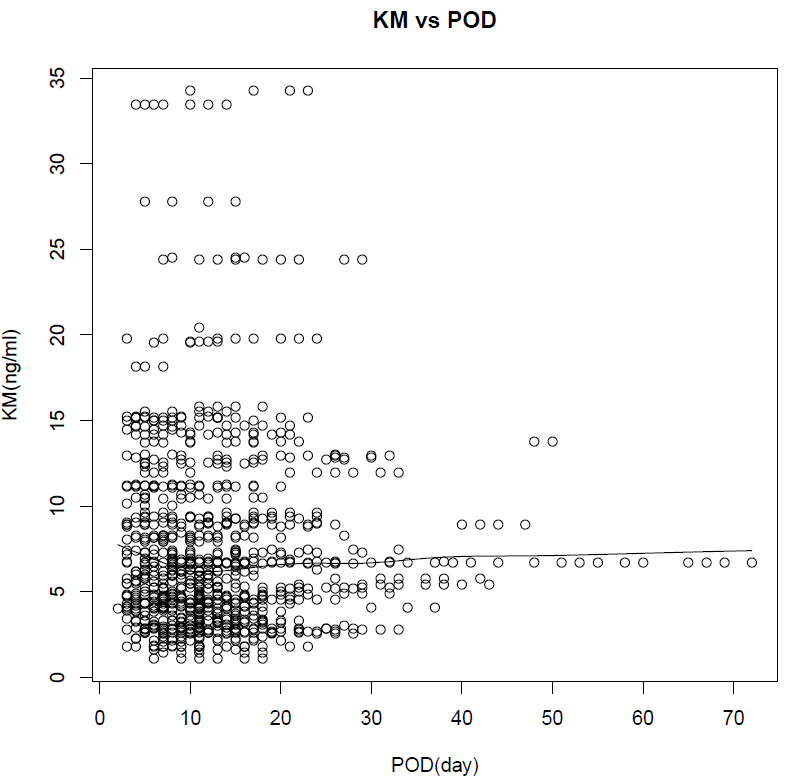


**Figure S1 The correlations between the Km and POD in the MM model.** The K_m_ (y-axis) showed a clear trend when plotted against the POD (x-axis). K_m_ denotes the steady-state concentrations at half-maximal dose rate; POD, postoperative days.
